# Corticosteroid transdermal delivery significantly improves arthritis pain and functional disability

**DOI:** 10.1007/s13346-016-0340-9

**Published:** 2016-12-07

**Authors:** Tommaso Iannitti, Michael F. McDermott, Carmen Laurino, Andrea Malagoli, Beniamino Palmieri

**Affiliations:** 1 KWS BioTest, Marine View Office Park, Portishead, Somerset BS20 7AW UK; 2 Poliambulatorio del Secondo Parere, 41100 Modena, Italy; 3 National Institute for Health Research – Leeds Musculoskeletal Biomedical Research Unit (NIHR-LMBRU) and Leeds Institute of Rheumatic and Musculoskeletal Medicine (LIRMM), Wellcome Trust Brenner Building, St James’s University Hospital, Beckett Street, Leeds, West Yorkshire LS9 7TF UK; 4 Department of General Surgery and Surgical Specialties, University of Modena and Reggio Emilia Medical School, Surgical Clinic, 41100 Modena, Italy; 5 Department of Medical and Surgical Sciences for Children and Adults, University of Modena and Reggio Emilia, 41100 Modena, Italy

**Keywords:** Betamethasone valerate, Transdermal drug delivery, Pain, Functional disability, Stiffness, C-reactive protein, Arthritis, Osteoarthritis

## Abstract

Arthritis is characterized by pain and functional limitation affecting the patients’ quality of life. We performed a clinical study to investigate the efficacy of a betamethasone valerate medicated plaster (Betesil) in improving pain and functional disability in patients with arthritis and osteoarthritis. We enrolled 104 patients affected by osteoarthritis (*n* = 40) or arthritis (*n* = 64) in different joints. Patients received diclofenac sodium cream (2 g, four times a day) or a 2.25-mg dose of Betesil applied to the painful joint every night before bedtime for 10 days. Pain and functional disability were assessed, by the Visual Analogue Scale (VAS) and Western Ontario McMaster Universities Osteoarthritis Index (WOMAC) scores. Redness was assessed by clinical inspection, and edema by the “fovea sign” method. C-reactive protein (CRP) was also measured; CRP can be used to cost-effectively monitor the pharmacological treatment efficacy and is increased during the acute-phase response, returning to physiological values after tissue recovery and functional restoration*.* All measurements were at baseline and at 10-day follow-up. At 10-day follow-up, a greater improvement in VAS and WOMAC pain and WOMAC stiffness and functional limitation scores from baseline was observed in patients treated with Betesil compared with diclofenac (all *p* < 0.01). At 10-day follow-up, improvement in redness, edema, and CRP levels from baseline was also greater in patients treated with Betesil compared with diclofenac (all *p* < 0.01). This study demonstrates the safety and efficacy of transdermal delivery of betamethasone valerate in patients affected by arthritis and osteoarthritis.

## Introduction

Arthritis is a pathological condition that causes pain and inflammation in a joint. Osteoarthritis (OA) is the most common form of arthritis and is defined as a degenerative mesenchymal disease affecting an estimated 10 % of the world’s population over 60 years [1, 2]. Symptoms include pain, stiffness, and functional limitation, leading to loss of autonomy and poor quality of life [3]. Various treatment options are available for OA management. These include (1) non-steroidal anti-inflammatory drugs (NSAIDs) for pain management [4], (2) bisphosphonates to decrease pain and improve functionality by preserving the structural integrity of subchondral bone [5], (3) pulsed electromagnetic field therapy [6], and (4) viscosupplementation, with hyaluronic acid alone or in combination with bisphosphonates or NSAIDs, to improve pain and functional activity [7–9]. Indeed, viscosupplementation with hyaluronic acid improves articular cartilage degeneration and decreases osteophyte formation, as shown by experimental studies using OA models [10, 11]. Topical corticosteroids also decrease pain and improve joint functionality [12, 13]. The non-pharmacological management of OA includes education and self-management, exercise and weight loss, assistive devices, alternative and complementary approaches, and surgical interventions [14].

### Transdermal delivery of betamethasone valerate for treatment of arthritis

So far, only a few studies have investigated the clinical efficacy of transdermal delivery of corticosteroids for OA management. Corticosteroids for the treatment of arthritis may be administered by iontophoresis, a non-invasive technique that allows transdermal drug delivery. Betamethasone valerate (BMV) is a synthetic, moderately active corticosteroid without mineralocorticoid properties, [15] which binds the intracellular cytoplasmic glucocorticoid receptor and translocates it to the nucleus to function as a ligand-activated transcription factor [16]. Eight sessions of dexamethasone iontophoresis have been used for treatment of juvenile idiopathic arthritis affecting the temporomandibular joint [17]. Resolution of pain occurred in 73 % of the patients who had pain at baseline. On the other hand, a second study, evaluating the efficacy of a transdermal steroid delivery vs. placebo by iontophoresis or phonophoresis for the treatment of patients with trapeziometacarpal arthritis, did not show significant differences between treatments [18].

The aim of this study was to compare the efficacy of transdermal BMV and diclofenac sodium cream in patients affected by arthritis and OA, in order to determine the best therapeutic option in terms of pain, redness, edema, and functional disability.

## Materials and methods

This study was performed at the Poliambulatorio del Secondo Parere clinic (Modena, Italy) in accordance with the Declaration of Helsinki and approved by the Institutional Review Board at Poliambulatorio del Secondo Parere (Modena, Italy). All patients signed the informed consent and agreed to data collection and review.

### Patients’ demographics and disease characteristics

A total of 104 patients (62 males and 42 females) participated in this study. Originally, 106 patients were recruited but two patients, affected by hip arthritis, dropped out soon after recruitment due to the development of a symptomatic herniated disc and excluded from data analysis. Inclusion criteria were symptomatic arthritis and OA with a Visual Analogue Scale (VAS) pain score ≥ 60 mm. Exclusion criteria were hypersensitivity or allergy to the active component of the plaster, presence of skin edema, and joint effusions. Patients had a mean age of 57.3 ± 1.09 [mean ± standard error of the mean (SEM)]. Patients were diagnosed on the basis of medical examination and orthopedic evaluation using ultrasound, radiography, and magnetic resonance imaging as follows: bilateral knee OA (*n* = 24), hip arthritis (*n* = 4), OA with bulging disc between L4 and L5 (*n* = 16), sciatic nerve inflammation deriving from spinal arthritis occurring at L4, L5, and S1 (*n* = 20), shoulder arthritis (*n* = 28), and carpal tunnel syndrome associated with arthritis affecting the wrist (*n* = 12) (Table 1). All patients signed the informed consent.

**Table 1:** Patients’ demographics and disease characteristics at baseline and 10-day follow-up

	Diclofenac sodium cream (*n* = 52)	Betesil (*n* = 52)	*p*
Age	57.06 ± 1.52	57.54 ± 1.6	0.958
Female patients	18 (35 %)	24 (46 %)	0.318
Male patients	34 (65 %)	28 (54 %)	
Arthritis (non-rheumatoid)	33 (63 %)	31 (60 %)	0.84
Osteoarthritis	19 (37 %)	21 (40 %)	
Carpal tunnel syndrome/wrist arthritis	7 (13 %)	5 (10 %)	0.902
Hip arthritis	2 (4 %)	2 (4 %)	
Knee osteoarthritis	10 (19 %)	14 (27 %)	
Osteoarthritis with bulging disk (L4–L5)	9 (17 %)	7 (13 %)	
Sciatic nerve inflammation/spinal arthritis	9 (17 %)	11 (21 %)	
Shoulder arthritis	15 (29 %)	13 (25 %)	
VAS (mm; baseline)	75 (70–80)	87 (78–91.25)	< 0.01
VAS (mm; follow-up)	65 (60–70)	35 (32–43.25)	< 0.01
WOMAC (pain; baseline)	14 (10–17)	15 (10–19)	0.384
WOMAC (pain; follow-up)	12 (9–16)	7 (4–10)	< 0.01
WOMAC (functional limitation; baseline)	51.5 (40.75–60)	51 (39–61.25)	0.706
WOMAC (functional limitation; follow-up)	47 (38.5–55)	25.5 (17–39.25)	< 0.01
WOMAC (stiffness; baseline)	5 (4–7)	6 (4.75–8)	0.083
WOMAC (stiffness; follow-up)	4.5 (3–6)	3 (2–4.25)	< 0.01
CRP (mg/l; baseline)	4.25 (2.98–6.03)	4.75 (3–6)	0.848
CRP (mg/l; follow-up)	3.65 (2.1–5.73)	2.25 (1–3)	< 0.01
Redness (baseline; score = 0)	0 (0 %)	0 (0 %)	0.32
Redness (baseline; score = 1)	9 (17 %)	15 (29 %)	
Redness (baseline; score = 2)	19 (37 %)	14 (27 %)	
Redness (baseline; score = 3)	24 (46 %)	23 (44 %)	
Redness (follow-up; score = 0)	0 (0 %)	21 (40 %)	< 0.01
Redness (follow-up; score = 1)	17 (33 %)	18 (35 %)	
Redness (follow-up; score = 2)	15 (29 %)	13 (25 %)	
Redness (follow-up; score = 3)	20 (38 %)	0 (0 %)	
Edema (baseline; score = 0)	0 (0 %)	0 (0 %)	0.189
Edema (baseline; score = 1)	12 (23 %)	12 (23 %)	
Edema (baseline; score = 2)	28 (54 %)	20 (38 %)	
Edema (baseline; score = 3)	12 (23 %)	20 (38 %)	
Edema (follow-up; score = 0)	0 (0 %)	23 (44 %)	< 0.01
Edema (follow-up; score = 1)	20 (38 %)	23 (44 %)	
Edema (follow-up; score = 2)	21 (40 %)	6 (12 %)	
Edema (follow-up; score = 3)	11 (21 %)	0 (0 %)	

### Study design

Patients were matched on the basis of age, sex, and type of pathology (*p* = 0.958, *p* = 0.318, and *p* = 0.84, respectively; Table 1). Patients were instructed to apply a 2.250-mg BMV medicated plaster (Betesil; *n* = 52) on the painful joint every night before bedtime for 10 days or diclofenac sodium cream (*n* = 52; 2 g) on the painful joint four times a day for 10 days. If pain relief was not adequate, patients were instructed to take diclofenac sodium orally as rescue medication (50 mg, up to three capsules per day).

### Pain and functional disability assessment

VAS (0–100 mm; 0 mm = minimum pain; 100 mm = maximum pain) and Western Ontario McMaster Universities Osteoarthritis Index (WOMAC) scores were used to evaluate the efficacy of Betesil on pain and functional disability at 10-day follow-up. WOMAC is based on five items related to pain (subscore 0–20; 0 = minimum pain; 20 = maximum pain), two to stiffness (subscore 0–8; 0 = minimum stiffness; 8 = maximum stiffness), and 17 to functional limitation (subscore 0–68; 0 = minimum functional limitation; 68 = maximum functional limitation).

### Redness and edema assessment

Redness was assessed by visual clinical inspection (subscore 0–3; 0 = absence of redness, 1 = slight redness, 2 = moderate redness, and 3 = intense redness). Edema was assessed by the “fovea sign” method [19]. The fovea sign is positive when there is exquisite tenderness compared with the contralateral side and is scored as follows: subscore 0–3, 0 = absence of edema, 1 = 2-mm edema following depression of the skin, 2 = 4-mm edema following depression of the skin, and 3 = 5-mm edema following depression of the skin.

### C-reactive protein

The venous blood of all fasting subjects was drawn in the morning, and C-reactive protein (CRP) levels were detected by immunoturbidimetric method, using an automated analyzer. CRP has been extensively used in the orthopedic and rheumatology clinics [20, 21]. CRP is increased during the acute-phase response while it returns to physiological values following tissue recovery and functional restoration [22]*.* Therefore, in this study, CRP was used to allow cost-effective monitoring of the pharmacological treatment efficacy.

### Statistical analysis

Patients’ age is presented as mean ± SEM; VAS, WOMAC, and CRP data are reported as median [interquartile range (IQR)]; edema and redness data are presented as frequencies (percentages) (Table 1). Comparison of age between groups was performed using Student’s *t* test. Comparison of post-treatment percentage variation from baseline for VAS, WOMAC, and CRP data was performed using Wilcoxon’s signed-rank test. Comparison of post-treatment percentage variation for edema and redness data from baseline was performed using Pearson’s chi-square test. A *p* value < 0.05 was considered significant. All statistical analyses were conducted using R software [23].

## Results

**Fig. 1:**
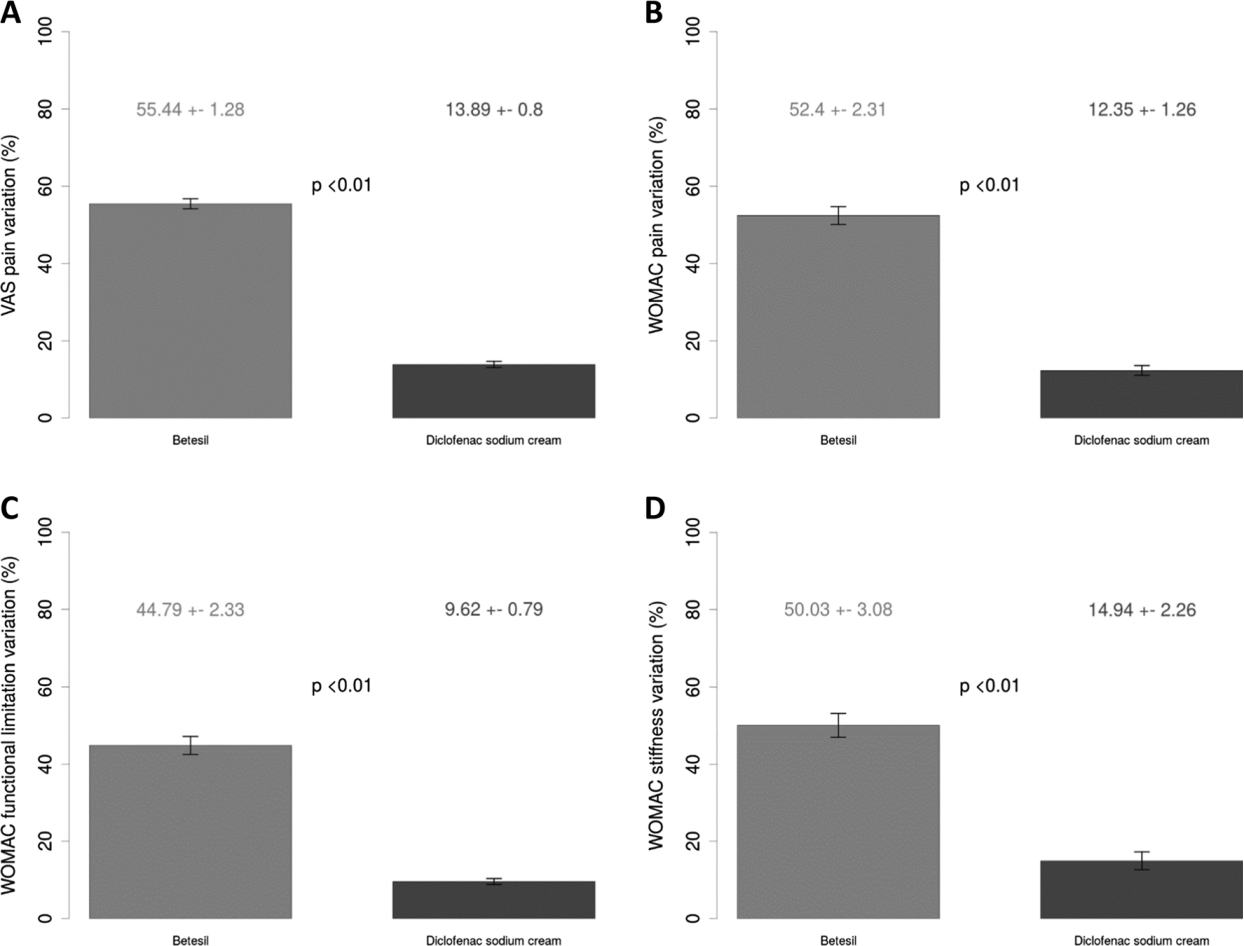
Betesil improves arthritis and osteoarthritis pain. Percentage variation in VAS pain (**a**), WOMAC pain (**b**), WOMAC functional limitation (**c**), and WOMAC stiffness (**d**) scores from baseline at 10-day follow-up following Betesil and diclofenac sodium cream treatment in patients affected by arthritis and osteoarthritis affecting different joints

**Fig. 2:**
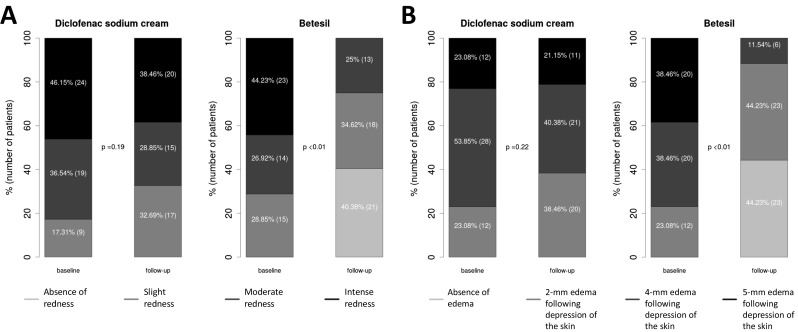
Betesil improves redness and edema scores in arthritis and osteoarthritis patients. Percentage variation in redness (**a**) and edema (**b**) from baseline at 10-day follow-up following Betesil and diclofenac sodium cream treatment in patients affected by arthritis and osteoarthritis affecting different joints

**Fig. 3:**
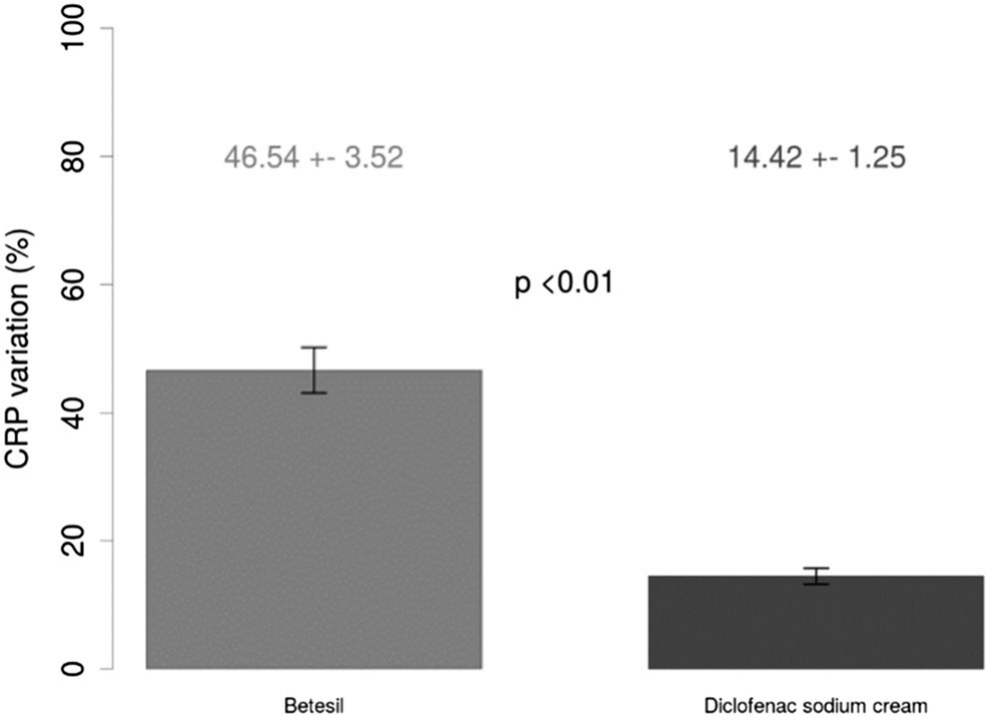
Betesil improves CRP in arthritis and osteoarthritis patients. Percentage variation in CRP from baseline at 10-day follow-up following Betesil and diclofenac sodium cream treatment in patients affected by arthritis and osteoarthritis affecting different joints

Table 1 shows baseline and 10-day post-treatment patients’ characteristics. At 10-day follow-up, a greater reduction in VAS (Fig. 1a) and WOMAC pain (Fig. 1b) scores from baseline was observed in patients treated with Betesil (55.44 ± 1.28 % and 52.4 ± 2.31 %, respectively), when compared with diclofenac sodium cream (13.89 ± 0.8 % and 12.35 ± 1.26 %, respectively; all *p* < 0.01). A greater reduction in WOMAC functional limitation (Fig. 1c) and stiffness (Fig. 1d) scores from baseline was observed in patients treated with Betesil (44.79 ± 2.33 % and 50.03 ± 3.08 %, respectively), when compared with diclofenac sodium cream (9.62 ± 0.79 % and 14.94 ± 2.26 %, respectively; all *p* < 0.01). A similar trend was observed for redness (Fig. 2a) and edema (Fig. 2b) scores in patients treated with Betesil, when compared with diclofenac sodium cream (all *p* < 0.01). A greater reduction in CRP levels (Fig. 3) from baseline was also observed in patients treated with Betesil (46.54 ± 3.52 %), when compared with diclofenac sodium cream (14.42 ± 1.25 %; *p* < 0.01). During the study, diclofenac sodium was used by 15 patients; 10 patients took two capsules a day for 2 days and then a capsule a day for 2 days during the first 4 days of plaster application. A total of five patients took a capsule a day for 2 days, during the first 2 days of plaster application. Only one patient reported a skin rash 4 hours after the first application of the plaster. The rash was thought to be an allergic reaction and resolved without medication after 5 hours. This patient continued applying the plaster the following days and completed the study.

## Discussion

To the best of our knowledge, this is the first demonstration of the clinical effectiveness and safety of a BMV medicated plaster for treatment of symptomatic joint arthritis and OA. Scientific interest in transdermal drug delivery systems has significantly increased in the last two decades because this approach is considered a valid therapeutic alternative to oral and more invasive strategies. The advantages of transdermal drug delivery include better patient compliance, control over input kinetics, and a lower incidence of gastrointestinal-related side effects [24], as observed in this study. Furthermore, it avoids the first-pass hepatic metabolism and plasma bioavailability fluctuations usually observed with oral administration [25]. In conclusion, the present study shows that Betesil is well tolerated and displays superior efficacy in reducing pain and functional disability and ameliorating CRP levels, redness, and edema, when compared with diclofenac sodium cream, in patients affected by arthritis and OA. The improvement in CRP levels supports tissue recovery and functional restoration [22] allowing us to monitor the therapeutic efficacy of Betesil and further supporting its use as a local pharmacological therapy for arthritis pain and functional disability.

### Authors’ contributions

TI, MFM, CL, AM, and BP conceived the study, drafted the manuscript, participated in the design of the study and data collection, and performed the statistical analysis. BP coordinated the study. All authors read and approved the final manuscript.
